# Semantic ambiguity effects on traditional Chinese character naming: A corpus-based approach

**DOI:** 10.3758/s13428-017-0993-4

**Published:** 2017-11-09

**Authors:** Ya-Ning Chang, Chia-Ying Lee

**Affiliations:** 1 Department of Psychology, Lancaster University, Lancaster, LA1 4YF UK; 2 Institute of Linguistics, Academia Sinica, Taipei, Taiwan

**Keywords:** Semantic ambiguity, Chinese character naming, Latent semantic analysis, Contextual diversity, Semantic diversity

## Abstract

Words are considered semantically ambiguous if they have more than one meaning and can be used in multiple contexts. A number of recent studies have provided objective ambiguity measures by using a corpus-based approach and have demonstrated ambiguity advantages in both naming and lexical decision tasks. Although the predictive power of objective ambiguity measures has been examined in several alphabetic language systems, the effects in logographic languages remain unclear. Moreover, most ambiguity measures do not explicitly address how the various contexts associated with a given word relate to each other. To explore these issues, we computed the contextual diversity (Adelman, Brown, & Quesada, *Psychological Science*, 17; 814–823, 2006) and semantic ambiguity (Hoffman, Lambon Ralph, & Rogers, *Behavior Research Methods*, 45; 718–730, 2013) of traditional Chinese single-character words based on the Academia Sinica Balanced Corpus, where contextual diversity was used to evaluate the present semantic space. We then derived a novel ambiguity measure, namely *semantic variability*, by computing the distance properties of the distinct clusters grouped by the contexts that contained a given word. We demonstrated that semantic variability was superior to semantic diversity in accounting for the variance in naming response times, suggesting that considering the substructure of the various contexts associated with a given word can provide a relatively fine scale of ambiguity information for a word. All of the context and ambiguity measures for 2,418 Chinese single-character words are provided as supplementary materials.

**Electronic supplementary material:**

The online version of this article (10.3758/s13428-017-0993-4) contains supplementary material, which is available to authorized users.

Words that can be associated with multiple meanings are *ambiguous*, because their exact use varies depending on the immediate language context. For example, *bank* can refer to a place where people can save or borrow money, but it can also refer to a raised piece of land along the side of a river. Each of these aspects of the meaning of *bank* can be considered a word sense. A number of psycholinguistic studies have examined the influences of semantic ambiguity on lexical decision (Azuma & Van Orden, 1997; Borowsky & Masson, 1996; Hino & Lupker, 1996; Hoffman & Woollams, 2015; Jastrzembski, 1981; Kellas, Ferraro, & Simpson, 1988; Millis & Bution, 1989; Rubenstein, Garfield, & Millikan, 1970), naming (Borowsky & Masson, 1996; Hino & Lupker, 1996; Lichacz, Herdman, Lefevre, & Baird, 1999; Rodd, 2004; Woollams, 2005), and semantic tasks (Hino, Pexman, & Lupker, 2006; Hoffman & Woollams, 2015; Pexman, Hino, & Lupker, 2004). We started by reviewing the ambiguity effects in lexical–semantic processing. In particular, we addressed how corpus-based semantic ambiguity measures could help resolve the limitations of the conventional subjective measures. We then proposed a novel ambiguity measure to try to improve the current ambiguity measures and tested its effects on traditional Chinese character naming.

## Semantic ambiguity effects in lexical–semantic processing

In lexical decision, an ambiguous benefit has been reported for words with multiple meanings. Ambiguous words are processed more accurately and quickly than unambiguous words. The ambiguous advantage effect is strong particularly when words are tested against word-like nonwords (Borowsky & Masson, 1996; Kellas et al., 1988); presumably, greater semantic information is required.

As compared to lexical decision, the effects of ambiguity on naming latencies appear to be less conclusive. Some studies have demonstrated an ambiguous advantage effect (Hino & Lupker, 1996; Lichacz et al., 1999; Woollams, 2005), whereas others have shown a weak (Rodd, 2004) or even a null effect (Borowsky & Masson, 1996). The discrepant findings might be because a potential confounding factor, spelling-to-sound consistency, is not always considered in previous studies (Woollams, 2005). It is evident that consistency is a critical factor that affects response latencies in naming (Glushko, 1979; Jared, 1997). Woollams found an interaction between ambiguity and consistency in which the ambiguous advantage was much stronger for inconsistent words relative to consistent words. This might explain the null effect reported by Borowsky and Masson because most items they used were consistent words. It would seem that most studies on naming and lexical decision tasks have reported a facilitatory effect for ambiguous words. However, it is worth noting that both tasks generally do not require precise semantic information. If, for example, the exact meaning of a given word needs to be accessed in a semantic-relatedness decision task, competition will occur among the multiple meanings for ambiguous words, resulting in an inhibition effect (Hoffman & Woollams, 2015). Collectively, these findings suggest that ambiguity is an important factor affecting our lexical–semantic processing.

Of particular relevance here is how our lexical-processing system benefits from the ambiguity of words in naming and lexical decision. Some studies have used number of discrete meanings in a dictionary as a measure of semantic ambiguity (Jastrzembski, 1981; Rodd, 2004; Rodd, Gaskell, & Marslen-Wilson, 2002). Others have asked participants to write down the first meaning (or all of the meanings) for a word or to judge whether a word has more than one meaning (Azuma & Van Orden, 1997; Borowsky & Masson, 1996; Kellas et al., 1988; Millis & Bution, 1989; Rubenstein et al., 1970). These approaches assume that the meanings associated with a word are discrete so ambiguity advantage could result from multiple separate lexical entries for ambiguous words in the system (Jastrzembski, 1981; Kellas et al., 1988). For example, Kellas et al. suggest that when ambiguous words are processed, the lexical entries associated with them would be active simultaneously, and other competitors would be inhibited, resulting in faster recognition. Alternatively, within the distributed representation view, the word form of an ambiguous word can be mapped onto multiple distinct but overlap semantic representations (Borowsky & Masson, 1996; Kawamoto, Farrar, & Kello, 1994). Although it seems that activation of multiple semantic representations might interfere with each other, Borowsky and Masson demonstrated that in a neural network model, ambiguous words had a benefit of having multiple finishing states that could overcome competitions between their semantic representations. Another possible explanation is that for ambiguous words in a neural network, the model has to learn the mappings between an orthographic form and its multiple semantic representations, so stronger weight connections are expected relative to unambiguous words, which have a one-to-one mapping from orthography to semantics (Kawamoto et al., 1994).

These interpretations of ambiguity advantage assume that multiple meanings associated with a word are adequately separate that correspond to either different lexical entries or distinct semantic representations. However, as was pointed out by Rodd, Gaskell, and Marslen-Wilson (2002), words can be ambiguous in more subtle and different ways depending on the relationships between word senses that the words have. For instance, according to the *Wordsmyth Dictionary–Thesaurus* (Parks, Ray, & Bland, 1998), *bank* has two lexical entries in which six senses are associated with financial institution meaning and another six senses are associated with sloping mound meaning. The relationship between the senses associated with the same meaning is *polysemy* whereas the relationship between the senses associated with different meanings is *homonymy*. Both homonymy and polysemy are possible sources of semantic ambiguity. The findings of ambiguity advantage reported by some previous studies (Azuma & Van Orden, 1997; Borowsky & Masson, 1996; Kellas et al., 1988) used words that only differed in the number of senses but not always in the number of meanings. When they considered the two types of ambiguity in lexical decision, Rodd, Gaskell, and Marslen-Wilson (2002) demonstrated that ambiguity between multiple senses could *facilitate* response latencies, whereas multiple meanings could *prolong* the latencies. In a subsequent study, Rodd, Gaskell, and Marslen-Wilson (2004) developed a computational model and demonstrated differential effects of ambiguity between meanings and between senses. In their model, ambiguous words were represented by two randomly generated semantic representations and to simulate few- or many-sense words, half of the words had noise added to the representations, and the other half did not. Unambiguous words were simulated in a similar manner except that the words were represented by only one random semantic representation. During training, large attractors were developed for unambiguous words with many senses because the model learned the mappings from one orthographic form to many related semantic representations. Thus when these words were presented, their semantic activations were quickly activated, which in turn facilitated the activations of their phonological codes and even the semantic activations were noisy. By contrast, for ambiguous words with few senses, relatively small attractors were developed for each unrelated semantic representation that could not effectively facilitate semantic activations; instead the competition between them resulted in an inhibition effect.

## Corpus-based semantic ambiguity measures

The measure of ambiguity based on dictionary definitions has some important limitations. For instance, the number of senses for a word defined in different dictionaries could vary widely. Also, the measure based on dictionary definitions could be potentially overestimated because participants generally cannot report most dictionary senses for a word (Gernsbacher, 1984). Moreover, several researchers have raised the fundamental question of whether the ambiguity of a word can be measured by a discrete number of senses or meanings (Hoffman, Lambon Ralph, & Rogers, 2013; Hoffman & Woollams, 2015). Whether two uses of a word should be considered as two unrelated senses (i.e., *homonymy*) or two related senses (i.e., *polysemy*) is not always well defined. To address this topic, a different view that considers diverse linguistic environments has been proposed. It takes into account that the use of a word is highly dependent on immediate linguistic contexts (Hoffman, Lambon Ralph, & Rogers, 2013; Landauer, 2001). According to this view, the diversity of the contexts where a word can appear provides the source of semantic ambiguity. The diversity of contexts associated with a given word could be quantified by conducting a corpus analysis (Adelman, Brown, & Quesada, 2006; Hoffman et al., 2013; Jones, Johns, & Recchia, 2012; McDonald & Shillcock, 2001). Adelman et al. derived a measure of *contextual diversity (CD)* by counting the number of documents (contexts) in a large text corpus that contains a given word. The CD has proven to be a better variable than word frequency in predicting response latencies in both lexical decision and naming (Adelman et al., 2006; Brysbaert & New, 2009; Keuleers, Brysbaert, & New, 2010). However, the CD has no intention of addressing variation in meanings across contexts that a given word is used (Hoffman & Woollams, 2015). Several studies have examined the effects of semantic ambiguity caused by contextual usage (Hoffman et al., 2013; Jones et al., 2012; McDonald & Shillcock, 2001). Hoffman et al. (2013) derived a variable termed *semantic diversity (SemD)* measuring the degree of dissimilarity between all the contexts that a word has been seen. If a word tends to appear in the similar contexts, the uses of that word are similar so the word is less ambiguous; by contrast, if a word can be used in diverse contexts then it is more ambiguous. This is to effectively measure the relatedness of word senses (or uses) associated with a given word. Recently, Hoffman and Woollams demonstrated that higher SemD words were processed faster relative to lower SemD words in the lexical decision tasks, which resembles the ambiguity advantage effect reported in Rodd et al. (2002). They proposed that SemD could be used as an alternative for quantifying polysemy, in which variation in a word’s meaning would change continuously with various linguistic contexts. Other related measures, such as *semantic distinctiveness* (Jones et al., 2012), measure the degree of overlap between all the documents containing a given word.

The existing studies on corpus-based ambiguity measures have demonstrated that the ambiguity effect could originate from variation in contextual usage. However, according to Hoffman et al. (2013), SemD could not adequately differentiate words (e.g., *bark*) that have both homonymous and polysemous senses from words (e.g., *chance*) that have only polysemous senses and it would assign similar high SemD scores to them. The difference between homonymous and polysemous senses might be reflected in the substructure underlying variation in contextual usage of the words but this has not been addressed in Hoffman et al. (2013). However, the fundamental question is whether the substructure of word usage is important to lexical-semantic processing. More importantly, whether the ambiguity measures based on contextual variation can to some degree provide information about the interrelationships among different uses of words.

## Semantic ambiguity effects on Chinese character processing

Although the ambiguity measures based on contextual variation has proved to be important to lexical–semantic processing and can serve as useful alternatives to traditional ambiguity measures, it remains unclear whether this could be generalized to different language systems, especially for languages that use a logographic writing system such as Chinese. In Chinese, the basic orthographic writing unit is the character. Most common Chinese characters are free morphemes that can be used alone in the text and carry useful semantic information, also known as *single-character words*. Additionally, there are two types of Chinese scripts: traditional and simplified. They are used in different Chinese speaking regions (the traditional script for Taiwan and Hong Kong; the simplified script for Mainland China, Singapore, and Malaysia). Simplified characters are created from traditional characters using a series of simplification processes (McBride-Chang, Chow, Zhong, Burgess, & Hayward, 2005). For examples, 請→请 /qing3/ and 華→华 /hua2/. The simplification process is applied to about 33.57% of original traditional Chinese characters (Liu, Chuk, Yeh, & Hsiao, 2016), which means a considerable portion of characters are still shared between two scripts. Despite the obvious difference between English and Chinese in their orthographic writing systems, most typical reading effects such as frequency effects (e.g., Forster & Chambers, 1973; Lee, Tsai, Su, Tzeng, & Hung, 2005), regularity, or consistency effects (e.g., Glushko, 1979; Lee et al., 2005) have a similar pattern across English and Chinese. A number of studies in Chinese also have shown ambiguity advantages in naming and lexical decision (Chang, Hsu, Tsai, Chen, & Lee, 2016; Lee, Hsu, Chang, Chen, & Chao, 2015; Peng, Deng, & Chen, 2003; Sze, Yap, & Rickard Liow, 2015; although see Liu, Shu, & Li, 2007, for a null effect), in which response times (RTs) are faster for ambiguous than for unambiguous characters. Take the character 花 /hua1/ as an example of an ambiguous character. It can refer to a flower, spending money, a state of bloom, or something multicolor. Another example is 生 /sheng1/. It has different meanings such as raw, living, grow, giving birth, and starting a fire. The exact use of these ambiguous words depends on their immediate language contexts. By contrast, some characters are relatively unambiguous; for example, 鉀 /jia3/ refers to potassium, and 糖 /tang2/ refers to sugar. Similar to the results from studies in English, most ambiguity measures used in Chinese are based on either dictionary definitions or subjective ratings of the number of meanings (Hsu, Lee, & Marantz, 2011; Liu et al., 2007). The effect of the semantic ambiguity of Chinese characters has not yet been considered in light of the characters’ contextual variation.

The present study was set up to address two main issues: (1) whether the corpus-based approach of semantic ambiguity is applicable to Chinese language processing; (2) whether considering the substructure underlying variation in meaning and contextual usage could add valuable information to the existent ambiguity measures, and could make a link to the concepts of homonymy and polysemy. We focused on investigating Chinese characters that have an independent meaning (i.e., single-character words) because they are the smallest word segmentation unit in the text corpus and are comparable to monosyllabic words in English (Liu et al., 2007). First, we derived contextual diversity (Adelman et al., 2006) based on the Academia Sinica Balanced Corpus (ASBC; Huang & Chen, 1998) to test whether it is a strong predictor in naming. The effect of contextual diversity on naming in Chinese has been reported by Cai and Brysbaert (2010) on the basis of film subtitles. The effect also has been recently demonstrated in the Chinese lexical decision task (Sze, Rickard Liow, & Yap, 2014; Sze et al., 2015). Thus, this effect could be used to verify the semantic space we constructed on the basis of the ASBC, and to confirm the importance of contextual variation in Chinese word naming. Next, we investigated the ambiguity effect based on the corpus-based approach in Chinese character naming. We also sought to address the substructure underlying variation in meaning and contextual usage. We computed two semantic diversity measures: one was *semantic diversity*, which was adopted from Hoffman et al. (2013) and the other one was *semantic variability*, which was our novel application to address the degree to which the various contexts associated with a given word are similar in their general meaning, reflecting how the words are used and the interrelations among word senses. Specifically, we examined distance properties of the clusters grouped by those contexts associated with the words. It is assumed that conceptually distinct clusters can represent a discrete number of senses or meanings. In addition, how similarly two clusters (meanings) are related to each other can be quantified by measuring *between-group* distance and how similarly the contexts (senses) within the same cluster are related to each other can be quantified by measuring *within-group* distance. Word ambiguity can be considered as a continuum ranging from words with multiple distinct unrelated senses (homonymy) to words with multiple highly related senses (polysemy) but most of the words have both types of senses and the degree of relatedness between the senses is different. By combining both between-group and within-group distances, it is possible to characterize the continuum of ambiguity. As an example, for words having multiple relatively unrelated meanings, it is expected that the ambiguity can be characterized by more distinctly tight clusters (i.e., large between-group distance but small within-group distance). On the other hand, for words having multiple strongly related meanings, it is expected that the ambiguity can be characterized by looser clusters (i.e., small between-group distance but large within-group distance). The predictive power of the two ambiguity measures in Chinese character naming was compared.

## Method

### Chinese semantic space

Latent semantic analysis (LSA) is one of the important co-occurrence statistics that has been widely used in psycholinguistic studies across different languages, including English and Chinese (Chen, Wang, & Ko, 2009; Landauer & Dumais, 1997; Wang, Hsu, Tien, & Pomplun, 2014). LSA derives a semantic space based on a collection of segmented documents in which the number of occurrences of a word in various types of documents is computed as an element in the high-dimensional co-occurrence matrix. The dimensionality of the matrix is then reduced by using singular-value decomposition (SVD) that preserves the semantic relations between words as much as possible.

We implemented Chinese LSA based on the Academia Sinica Balanced Corpus (ASBC; Huang & Chen, 1998). The ASBC corpus consists of 9,227 documents with a total size of five million traditional Chinese words. The documents covered various fields including science, social society, art, lifestyle, philosophy, and literature. In the preprocessing of the corpus, numerical values, symbols, HTML tags, names, punctuation marks, and alphabetic letters were removed. All 9,227 documents were included for processing. Because some of the documents in the ASBC are very long (over thousands of words), following Hoffman et al. (2013), we subdivided each document into small chunks. Each chunk consisted of roughly 150 Chinese words as a separate context. We then performed a standard LSA procedure by creating a high-dimensional co-occurrence (i.e., word–context) matrix. Words were excluded if they were very high in frequency (top 3%) or appeared less than five times in the corpus. This resulted in a 31,170 × 34,565 dimensional matrix. Prior to reducing the dimensionality of the matrix, a logarithmic transformation was applied in order to reduce the great influence of very high-frequency function words in generating the semantic space (Hoffman et al., 2013; Landauer & Dumais, 1997). SVD was then used to reduce the high-dimensional matrix to 300 dimensions, which is the typical dimensionality for both English and Chinese semantic spaces (Chen et al., 2009; Landauer & Dumais, 1997).

### Contextual diversity and semantic ambiguity measures

Contextual diversity and two semantic ambiguity measures (semantic diversity and semantic variability) were computed on the basis of the Chinese LSA space. We focused on single-character words rather than multicharacter words because most available large-scale naming data that can be used to verify our measures were based on a single character (Chang et al., 2016; Liu et al., 2007). The present Chinese LSA space contained 2,418 single-character words, which were free morphemes. The detailed procedures for generating different measures are described below.

#### Contextual diversity *(CD)*

**Fig. 1:**
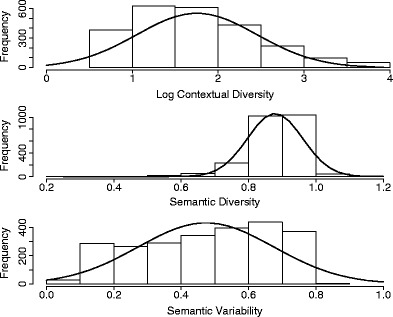
Distributions of single-character words as a function of log contextual diversity (upper panel), semantic diversity (middle panel), and semantic variability (lower panel), with their normal distribution curves

The CD score was derived by counting the number of contexts associated with a given word. This followed the definition by Adelman et al. (2006). Higher scores indicated that a word could be used in more diverse contexts. The scores ranged from 6 to 8,386. After log-transforming the values, the distribution of characters as a function of log CD is illustrated in the upper panel of Fig. 1.

#### Semantic diversity *(SemD)*

Hoffman et al. (2013) counted the number of documents (contexts) for a given word in the British National Corpus by using LSA. They then computed the average distance of all the pairwise contexts that contained the word. Following Hoffman et al. (2013), we computed SemD on the basis of the semantic vectors generated for contexts in the LSA space. For each word, we recorded all the contexts where it appeared at least once in the LSA semantic space. If there were more than 2,000 contexts, the most frequent 2,000 contexts were used for further analyses. This could make the process traceable (Hoffman et al., 2013). For each word, the average cosine value between any two contexts containing that word was calculated. The score was log-transformed, and the sign was reversed to represent the ambiguity. The scale of SemD ranged from 0.2507 to 1.1838. For a given word, a higher score indicates that the word is more semantically diverse. The distribution of characters as a function of SemD is illustrated in the middle panel of Fig. 1.

#### Semantic variability *(SemVar)*

The SemD measure proposed by Hoffman et al. (2013) considers how the various contexts associated with a given word relate to each other by taking the average of all the pairwise similarity scores. That is an intuitive way of looking at how the contexts are related to each other. However, the substructure underlying the contexts could not be fully addressed. For example, the issues raised are whether the contexts associated with a given word can be subdivided into different distinct groups of contexts, reflecting a distinct number of senses or meanings, and if so, what are the distance properties of those distinct clusters? We sought to resolve these issues by deriving a novel measure, *semantic variability*. We applied a *k*-means clustering technique (Kintigh, 1990; Kintigh & Ammerman, 1982) to examine the cluster structure of the context vectors. The *k*-means clustering algorithm is a data-driven method to partition a dataset into a number of groups, by minimizing the distance within clusters while maximizing the distance between clusters (Kintigh, 1990; Kintigh & Ammerman, 1982). For each word, we performed the *k*-means algorithm on the sets of context vectors containing it, and the best number of clusters was obtained. The context vectors were obtained from the LSA space described in the previous section, and the dimensionality of each context vector was 300. After the clusters were identified, we computed the average within-group distance and the average between-group distance of those clusters. To combine the between- and within-group distance scores, we divided the within-group distance by the between-group distance. The resulting score was used as a measure of semantic variability.

**Fig. 2:**
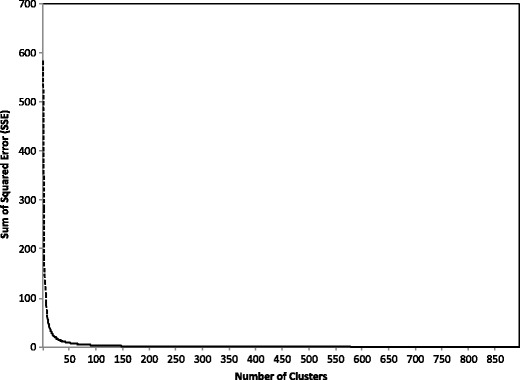
Within-group sum of squared errors (SSE) against number of cluster solutions for the single-character word 花

One complication of using the *k*-means algorithm was that the number of clusters must be specified initially. If an incorrect number of clusters had been selected, the partitions might be unreliable. A conventional method to tackle this issue is to perform the *k*-means algorithm with different number of clusters (Everitt, Landau, Leese, & Stahl, 2011; Peeples, 2011). The best number of clusters can be decided by looking for a bend in the sum of squared errors (SSE) plot against cluster solutions. SSE measures the distance between a cluster member and its cluster centroid and the error score generally decreases with the increase in number of clusters. In the present LSA semantic space, some words are highly contextually diverse and can appear in several thousands of contexts. However, our pilot explorations showed that the bends in the SSE plots for those contextual diverse words would seem to occur within hundreds of cluster solutions. This suggested that the large cluster solution did not greatly improve the total SSE so the range of cluster solutions could be kept within a reasonable length. For example, the single-character word, 花, has 895 contexts. Figure 2 shows the plot of the SSE against all possible cluster solutions for this word (i.e., from 1 to 895). As can be seen, the SSE decreases rather rapidly, and the solutions for numbers of clusters greater than 200 have a small impact on the SSE. Hence, for all the words, we performed the *k*-means algorithm and compared the SSE for up to 200 cluster solutions.^1^ The best number of clusters for each word was then decided by finding at which point there was a reduction of 90% of the SSE. The results showed that the best number of clusters ranged from 3 to 10 (*M* = 7.03, *SD* = 1.63), the scores for within-group distance ranged from .0073 to .583, and the scores for between-group distance ranged from .5197 to .9397; hence, the total SemVar scores ranged from .0136 to .8081. A higher score indicated that the level of variability in all of the contexts associated with a given word was higher. The distribution of words as a function of SemVar is illustrated in the lower panel of Fig. 1.

### Analyses

A series of linear mixed-effect models (LMM) was conducted. Models were fit using the lme4 package in R (version 3.2.0, 2015). As demonstrated by Cai and Brysbaert (2010), CD could contribute an additional variance in predicting Chinese naming RTs above and beyond frequency. To verify the semantic space we constructed here, we tested and compared the predictive power of CD and frequency to see if we could find a similar effect. Note that the frequency of the single-character words can be measured in two different ways (Cai & Brysbaert, 2010; Liu et al., 2007). The first is to look at the frequency of the occurrence of the characters regardless they are used as single-character words or as constituent characters in multi-character words, termed as *character frequency*. Another way is to measure the frequency of the occurrence of the characters only when they are used as single-character words, termed *word frequency*. In the present study, we used character frequency as the primary frequency measure of the single-character words because it has been shown to be a stronger predictor of naming than is word frequency (Cai & Brysbaert, 2010).

We then focused our attention on examining the relationships between SemD, SemVar, and other lexical-semantic factors that have previously been shown to be important in naming including character frequency, number of strokes, consistency, imageability, and in particular semantic ambiguity rating (Chang et al., 2016; Lee et al., 2005; Liu et al., 2007). We also tested the predictive power of all of the subjective and objective ambiguity measures in accounting for naming RTs given those lexical–semantic factors plus initial phonemes.

The initial phoneme of each character was coded dichotomously (1 or 0) for the following 13 features, where 1 denoted the presence of the feature and 0 denoted its absence: stop, affricate, fricative, nasal, liquid, aspirated, voiced, bilabial, labiodental, alveolar, palato-alveolar, alveolo-palatal, and velar. Character frequency (CF) was based on the number of occurrences in the ASBC corpus. Number of strokes (NoS) for a character was used as a measure of visual complexity of that character. Consistency (Cons) used here was based on the ratio of the summed frequencies of characters sharing a phonetic radical that had the same pronunciation, to the summed frequencies of characters sharing that phonetic radical (Lee et al., 2005). Both imageability (Img) and semantic ambiguity rating (SemR) were based on subjective ratings (Hsu et al., 2011; Liu et al., 2007). Most of the measures (i.e., character frequency, number of strokes, consistency, and semantic ambiguity rating) were taken from the norms based on traditional Chinese characters (Chang et al., 2016). But the imageability scores were based on simplified Chinese characters (Liu et al., 2007). Despite the fact that the orthographic forms are different in the traditional and simplified scripts, given that the meanings of both scripts are shared, it is anticipated that the imageability measure (addressing how easily a mental image could be aroused by a given character) derived from one script is applicable to the other. As we shall see later in the LMM results, imageability can account for a significant portion of variance in naming RTs.

Naming RTs were taken from the traditional Chinese naming database by Chang et al. (2016). The naming data included RTs for 3,314 characters that were obtained from 140 participants. Each participant received one of seven subsets of characters, so there were 20 responses per item. Incorrect responses and items that did not have all of the scores were removed from the analysis. In all, 1,000 characters remained, all of which were single-character words. There were a total of 18,539 observation points. A further 1.51% of the observations, which were outliers (greater than two standard deviations from the means), were discarded from the LMM analyses. Naming RTs and predictors with a right-skewed distribution including frequency and CD were log-transformed. All variables were scaled.

## Results

### Contextual diversity and frequency effects

The effects of log CD and log CF on naming RTs were assessed using the linear mixed-effects models. As a baseline, we constructed a baseline model, Model 0, with item and subject as random factors, and with log RTs as the dependent variable. When log CF was included as a fixed effect into the baseline model termed Model 1, it resulted in a significant improvement of model fit, *χ* ^2^(1) = 221.37, *p <* .001. Similarly, when log CD was included as a fixed effect into the baseline model termed Model 2, it also resulted in a significant improvement of fit, *χ* ^2^(1) = 227.44, *p <* .001. These results showed that both log CF and log CD were significant predictors in naming whereas the effect size of log CD might be slightly larger than that of log CF by looking at chi-square values. To test this observation, we conducted another model, Model 3 with fixed effects of both log CD and log CF in additional to random effects. We then computed the increase in Akaike information criterion (AIC) by contrasting Models 3 and 1 for log CD, and Models 3 and 2 for log CF. The results showed that the increase in AIC for log CF was 57, which was lower than that of log CD, which was 63, confirming log CD was a slightly better predictor than log CF in naming. The small difference between log CF and log CD is similar to the data reported by Cai and Brysbaert (2010). These results provide an effective evaluation of the semantic space that we created on the basis of Academia Sinica Balanced Corpus.

### Semantic ambiguity effects

#### Correlation and principal components analyses

To assess the reliability of the two corpus-based ambiguity measures (SemD and SemVar) that we had derived, we examined how the two measures were related to subjective semantic ambiguity ratings (SemR; Hsu et al., 2011) as well as their relationships with other most commonly used lexical–semantic predictors (i.e., log CF, NoS, Cons, and Img) by conducting a correlation analysis. Table 1 shows the correlations between all of these predictors. Both SemD and SemVar were significantly correlated with SemR (*r =* .238, *p <* .001, and *r =* .407, *p <* .001, respectively), showing that both the corpus-based and subjective ambiguity measures are relevant, presumably tapping into variation in meaning and contextual usage. We also found that all of the ambiguity measures were correlated with both log CF and Img, but to different degrees. To closely examine their relationships, we conducted a principal component analysis on all of these predictors. Table 2 shows the results of the principal component analysis with promax rotation.^2^ Three factors (eigenvalues greater than 1) were extracted from the analyses, which together accounted for 64% of the total variance. Factor 1 had high loadings on log CF, SemR, SemD, and SemVar. Factor 2 had high loadings on both Cons and NoS, and Factor 3 had high loadings on Img and SemVar. The three ambiguity measures all loaded strongly on the frequency factor but not on the other two, apart from SemVar, which also loaded highly on the imageability factor. Therefore, SemVar was the only measure that loaded highly on both the frequency and imageability factors. This might suggest that SemVar can capture both frequency and semantic aspects of lexical processing and may have a greater predictive power for naming RTs than the other two ambiguity measures.

**Table 1:** Correlations between predictors (except initial phonemes)

	Log CF	NoS	Cons	SemR	Img	SemD	SemVar
Log CF	1						
NoS	– .105^***^	1					
Cons	– .011	.127^***^	1				
SemR	.604^***^	– .167^***^	– .062^***^	1			
Img	– .222^***^	– .001	– .012	– .219^***^	1		
SemD	.257^***^	– .028^***^	– .097^***^	.238^***^	– .074^***^	1	
SemVar	.594^***^	– .130^***^	– .006^***^	.407^***^	.142^***^	.245^***^	1
Log CD	.610^***^	– .125^***^	– .052^***^	.403^***^	.134^***^	.166^***^	.959^***^

^***^Correlation is significant at the .001 level; ^**^Correlation is significant at the .01 level; ^*^Correlation is significant at the .05 level. Log CF: log character frequency; NoS: number of strokes; Cons: consistency; Img: imageability; SemR: semantic ambiguity rating; SemD: semantic diversity; SemVar: semantic variability

**Table 2:** Results of principal component analyses with promax rotation

	Factor 1	Factor 2	Factor 3
Log CF	**.88**	.10	– .13
NoS	– .06	**.68**	– .02
Cons	.12	**.82**	.00
SemR	**.74**	– .05	– .25
Img	– .03	– .01	**.95**
SemD	**.45**	– .09	– .04
SemVar	**.84**	.03	**.40**

Scores greater than .4 were marked in bold. Log CF: log character frequency; NoS: number of strokes; Cons: consistency; Img: imageability; SemR: semantic ambiguity rating; SemD: semantic diversity; SemVar: semantic variability

### LMM analyses

For testing the effects of SemD, SemVar, and SemR on naming RTs, we started by conducting a simple LMM model in which each ambiguity measure was added into the baseline model separately as a fixed factor. The baseline model included random effects of both items and subjects, and log RT was used as the dependent variable. Adding SemD to the model resulted in a significant improvement, *χ* ^2^(1) = 49.80, *p <* .001, and adding SemVar to the model also resulted in a significant improvement, *χ* ^2^(1) = 270.45, *p <* .001. A similar effect was also found for SemR, *χ* ^2^(1) = 139.65, *p <* .001. These results show that all three of the semantic ambiguity measures were reliable predictors in the naming task.

For testing the partial effects of the ambiguity measures, we constructed a full LMM model with random effects of items and subjects and fixed effects of all the lexical variables, including initial phonemes, log CF, NoS, Cons, SemR, Img, SemD, and SemVar. The LMM results are summarized in Table 3. One of the initial phonemes, Liquid, was highly correlated with other phonemes, so it was removed during the fitting process. An effect can be considered significant at the *p <* .05 level if the *t* value is well above 2 (Baayen, 2008). The results showed that the onset effects were significant in naming, which is consistent with the previous literature (Chang et al., 2016; Liu et al., 2007). Importantly, all of the lexical–semantic variables contributed significantly in accounting for naming RTs. High-frequency words, visually simple words, high-imageability words, and words with consistent orthography-to-phonology mappings were named more quickly. In addition, the subjective ambiguity measure (SemR) and the two objective ambiguity measures (SemD and SemVar) all had unique predictive value in the model. The results suggest that words that can be used in more diverse contexts and have more meanings are processed more rapidly in the naming task.

**Table 3:** Linear mixed-effect model fitted to log RTs in naming (*R*-squared = 31.16%, *n* = 1,000)

	Estimated	Std. Err	*t*	Wald (2.5% to 97.5%)	Increase in AIC	*χ* ^2^(1)
Stop	– .205	.086	– 2.38	– .373 to – .036	–	–
Affricate	– .197	.092	– 2.14	– .377 to – .017	–	–
Fricative	– .114	.085	– 1.34	– .281 to .053	–	–
Nasal	.040	.060	0.66	– .078 to .159	–	–
Liquid	–	–	–	–	–	–
Aspirated	– .424	.075	– 5.65	– .570 to – .277	–	–
Voiced	– .129	.025	– 5.21	– .177 to – .080	–	–
Bilabial	.255	.093	2.75	.073 to .437	–	–
Labiodental	.343	.099	3.48	.150 to .536	–	–
Alveolar	.312	.086	3.62	.143 to .481	–	–
Palato-alveolar	.459	.099	4.64	.265 to .653	–	–
Alveolo-palatal	.462	.094	4.89	.276 to .647	–	–
Velar	.208	.091	2.29	.030 to .386	–	–
Log CF	– .120	.013	– 8.94	– .146 to – .093	75	77.02^***^
NoS	.040	.009	4.26	.021 to .058	16	18.04^***^
Cons	– .051	.009	– 5.57	– .069 to – .033	28	30.62^***^
SemR	– .042	.011	– 3.69	– .065 to – .020	11	13.51^***^
Img	– .083	.010	– 8.41	– .102 to – .063	66	68.37^***^
SemD	– .031	.009	– 3.32	– .049 to – .013	9	10.95^***^
SemVar	– .073	.012	– 6.05	– .096 to – .049	34	36.04^***^

All predictors were scaled. Log CF: log character frequency; NoS: number of strokes; Cons: consistency; Img: imageability; SemR: semantic ambiguity rating; SemD: semantic diversity; SemVar: semantic variability. ^***^The chi-square value is significant at the .001 level

To provide a complementary test of the predictive power of each variable, we conducted a series of LMM models to investigate the importance of the variables. We computed the increase in AIC when a target variable was withheld from the full LMM model and the significance of the change in model fit. A large increase in AIC is expected if a variable makes a substantial contribution to the model fit. All of the lexical–semantic variables except initial phonemes were removed from the full model separately. The AIC results and the chi-square statistic are shown in the last two columns of Table 3.

The most important predictor was log CF, with a large increase in AIC (75), followed by Img (66) and SemVar (34). The other variables—Cons (28), NoS (16), SemR (11), and SemD (9)—provided moderate improvements to the model fit. These analyses demonstrated that both of the ambiguity measures, SemVar and SemD, derived from large corpora were good predictors of naming RTs, but SemVar was superior to SemD in terms of predictive power.

### The relationships between SemVar and frequency related measures

The LMM results demonstrate that SemVar is a reliable psycholinguistic variable to account for naming latencies. The question is whether SemVar is unique from other frequency-related measures such as frequency and CD, given that this measure is heavily dependent on the contexts associated with words and that high-frequency words tend to be used in many and more diverse contexts than low-frequency words. On the basis of our preceding results in Tables 1 and 3, it is clear that SemVar was positively correlated with log CF, but SemVar remained a strong predictor even when log CF was considered. However, the relationship between SemVar and CD (measuring the number of contexts associated with a given word) has not been directly addressed. Thus, we first examined the correlation between SemVar and CD. The result showed that SemVar was significantly correlated with CD, *r* = .446, *p* < .001. This is consistent with the assumption that if words appear in more contexts, they tend to have multiple meanings and be more semantically ambiguous. Nevertheless, we also found that SemVar was even more highly correlated with CD after it was log-transformed, *r* = .959, *p* < .001, suggesting that the relationship between the two variables is not linear. This result also suggests that SemVar may carry the context information in addition to clustering information, because SemVar is computed on the basis of all the contexts associated with a given word. If this is true, we would expect that SemVar could account for unique variance in naming latencies above and beyond that accounted for by log CD. Moreover, given that log CF is also strongly correlated with both log CD and SemVar (Table 1), it makes sense to investigate all of these variables together. To assess the unique effect of SemVar, we conducted two additional LMM analyses. In one LMM analysis (LMM 1), log CF, log CD, and SemVar were loaded as predictors along with all the other variables described in the previous section, and naming latency was the dependent variable. The other LMM analysis (LMM 2) was the same as the first one, except that instead of SemVar we used the residuals of SemVar after log CD was partialed out, which we termed *SemVarRes*. Thus, SemVarRes and log CD were orthogonal. This was a very conservative test of SemVar, as it completely removed all influence of log CD from SemVar. The results along with the significance of the change in model fit for each variable are shown in Table 4. As can be seen, log CF, log CD, and SemVar were significant predictors. Note, however, that the direction of the effect of log CD was *opposite* to what was expected, showing that the higher the contextual diversity, the slower the naming response. Thus, the effect of log CD was unreliable, presumably because of the high correlations between log CF, log CD, and SemVar. When the shared variance between log CD and SemVar was removed, the correct pattern of log CD was observed. Importantly, in these two LMM analyses, both SemVar and SemVarRes predicted unique variance in naming latencies, providing strong evidence that the clustering information carried by either SemVar or SemVarRes is crucial and that the effect is beyond those of all other predictors. It is worth noting that when comparing the predictive power of log CF and log CD in LMM 2, the AIC was larger for log CF (84) than for log CD (20), suggesting that log CF was a stronger predictor in the full model.

**Table 4:** Linear mixed-effect model fitted to log RTs in naming, with log CF, log CD, SemVar, or SemVarRes along with the other psycholinguistic variables

	LMM 1			LMM 2		
	Estimated	*t*	*χ* ^2^(1)	Estimated	*t*	*χ* ^2^(1)
Stop	– .208	– 2.43	–	– .208	– 2.43	–
Affricate	– .197	– 2.16	–	– .197	– 2.16	–
Fricative	– .112	– 1.32	–	– .112	– 1.32	–
Nasal	.042	0.71	–	.042	0.71	–
Liquid	–	–	–	–	–	–
Aspirated	– .426	– 5.72	–	– .426	– 5.72	–
Voiced	– .127	– 5.18	–	– .127	– 5.18	–
Bilabial	.254	2.75	–	.254	2.75	–
Labiodental	.335	3.42	–	.335	3.42	–
Alveolar	.310	3.62	–	.310	3.62	–
Palato-alveolar	.451	4.58	–	.451	4.58	–
Alveolo-palatal	.455	4.85	–	.455	4.85	–
Velar	.200	2.21	–	.200	2.21	–
**Log CF**	**– .129**	**– 9.48**	**86.18** ^***^	**– .129**	**– 9.48**	**86.18** ^***^
**Log CD**	**.110**	**3.31**	**10.89** ^***^	**– .057**	**– 4.76**	**22.37** ^***^
NoS	.040	4.27	18.05^***^	.040	4.27	18.05^***^
Cons	– .051	– 5.59	30.79^***^	– .051	– 5.59	30.79^***^
SemR	– .042	– 3.67	13.36^***^	– .042	– 3.67	13.36^***^
Img	– .084	– 8.61	71.55^***^	– .084	– 8.61	71.55^***^
SemD	– .022	– 2.30	5.26^*^	– .022	– 2.30	5.26^*^
**SemVar**	**– .174**	**– 5.29**	**27.67** ^***^	–	–	–
**SemVarRes**	–	–	–	**– .049**	**– 5.29**	**27.67** ^***^

Log CF: log character frequency; NoS: number of strokes; Cons: consistency; Img: imageability; SemR: semantic ambiguity rating; SemD: semantic diversity; log CD: log-transformed contextual diversity; SemVar: semantic variability; SemVarRes: the residuals of semantic variability after partialing out log CD

## General discussion

Considerable evidence has shown that semantically ambiguous words are processed more quickly and accurately in naming (Hino & Lupker, 1996; Lichacz et al., 1999; Rodd, 2004; Woollams, 2005). Most studies adopted the ambiguity measures based on subjective ratings or dictionary definitions. In this study, we demonstrated that the corpus-based ambiguity measures that address variation in contextual usage were strong predictors in accounting for the Chinese naming performance. We first derived the log CD measure based on the ASBC corpus, which provides a source of contextual usage for each Chinese single-character word. Our LMM results showed that log CD could contribute unique variance above and beyond log CF in naming when each was used as predictors on their own, congruent with previous studies in Chinese (Cai & Brysbaert, 2010) also in alphabetic languages (Adelman et al., 2006; Brysbaert & New, 2009; Keuleers et al., 2010). This demonstrates that log CD is more informative than log CF but the difference is small, similar to the data reported by Cai and Brysbaert. However, when other psycholinguistic variables were included into the LMM analyses, log CF appeared to be a stronger predictor than log CD. This suggests that the inclusion of other variables may share the same variance with log CD, particularly the corpus-based semantic ambiguity measures. Given that the evidence in favour of using log CD in the Chinese naming performance is not strong, the present results lend support to Cai and Brysbaert’s argument that the more prevalent frequency measure might still be used.

More importantly, the present study aimed to investigate the ambiguity effect based on contextual variability. According to Hoffman et al. (2013), a word is less ambiguous if it consistently appears in similar contexts; by contrast, the word is more ambiguous if it can be used in diverse contexts. The relevance of the contexts associated with a given word is therefore imperative. Indeed, we found both the corpus-based ambiguity measures (SemD and SemVar) measuring the degree to which the contexts were related to each other were reliable predictors in the Chinese naming task. A word that had a high SemD or SemVar score was named faster than a word that had a low SemD or SemVar score. In addition, we also examined the relationship between SemVar and frequency related measures including log CF and log CD. The results show a convergent result, providing strong evidence to SemVar as an effective psychological measure of semantic ambiguity. It is worth noting that the results in Table 4 also further demonstrated that SemVar was a composite variable that combined both contextual diversity (log CD) and refined semantic variability (SemVarRes), which carries the key information about how various senses of a given word are interrelated. Thus SemVarRes can be used as a purer semantic measure of ambiguity than SemVar. Thus, we provide both the scores of SemVar and SemVarRes for each word in the supplement. Collectively, the present results are consistent with the findings reported by studies in English (Hoffman et al., 2013; Hoffman & Woollams, 2015; Schwanenflugel, Harnishfeger, & Stowe, 1988; Schwanenflugel & Shoben, 1983), suggesting semantic ambiguity is associated with variability of contexts and situations. This is particularly interesting in Chinese. Since all of the Chinese characters used here are free morphemes, and most of them are phonograms that consist of a semantic radical and a phonological radical, the semantic radical generally can provide the information about meanings. Even so, the meanings of those characters will still be ambiguous if they are associated with diverse contexts.

The LMM results demonstrated that SemVar was a stronger variable than SemD in predicting the Chinese naming performance. The main difference between SemD and SemVar was that SemVar could provide information about the degree in which the associated contexts were diverse at a finer level than SemD. In particular, SemVar carried structural information of the contexts revealing how the contexts were clustered into subgroups and the closeness within and between subgroups. The clusters of contexts can be considered as distinct senses or meanings that a given word has, reflecting by the uses of the word in different sets of similar contexts. But why the substructure information among contexts is important? One possible explanation is that the substructure information can reveal the different sources of ambiguity. Whether words are ambiguous between multiple distinct meanings or multiple related senses (or meanings) has been shown to have very different effects on lexical processing (Rodd et al., 2002). As in Rodd et al.’s (2002) Experiment 2, they showed that ambiguous words (e.g., *slip*) having two distinct meanings and each with multiple senses were processed slower than ambiguous words (e.g., *mask*) having only one meaning but with the matched total number of senses. This suggests that even though ambiguous words have the same number of senses, whether some of the senses are homonyms to represent separate meninges has influence on the processing. It seems that SemVar can provide such information and serve as a better indicator to characterize the continuum of ambiguity in word meaning than is SemD.

Some evidence can be used to support this argument. For example, SemVar can assign higher scores to words having only polysemous senses but lower scores for words having both homonymous and polysemous senses and consider these two types of ambiguous words differently, whereas SemD could not. That is, a character, like 律 /lu4/ can occur in two sets of different contexts, one set pertains to law and the other pertains to the name of the poetic form and each set has some sense variations. Its SemVar score is .1328 and SemD score is 0.9642. On the other hand, a character like 輕 /qing1/ can occur in a set of diverse contexts, all related to light. Both its SemD (0.9535) and SemVar (.7130) are quite high. This evidence suggests that it is important to capture different types of ambiguity. It may also imply that semantic representations for different types of ambiguity are different, consistent with Rodd et al. (2004). They demonstrated the differential ambiguity effects of polysemy and homonymy in a computational model in which the semantic representations of words with polysemous senses were implemented by a set of semantic representations that shared the same core activation pattern but varied in different degrees, whereas the semantic representations of words with homonymous senses were implemented by using completely different semantic representations. However, future studies will need to further test the difference between SemD and SemVar in a wider range of tasks such as lexical decision and semantic relatedness tasks.

Given that the corpus-based semantic ambiguity measures have proved to be good predictors in the Chinese naming task, and they were positively correlated with the subjective ambiguity rating, this approach is potentially useful for deriving ambiguity measures for Chinese disyllabic words. Because the number of Chinese disyllabic words is very large (e.g., approximately 22,351 words in the ASBC corpus), it is difficult and time-consuming to collect the measures based on subjective ratings. Also, no single dictionary may cover all the words, and different dictionaries tend to provide different numbers of meanings or senses for the same word. In addition, for cross-linguistic application, we have demonstrated that semantic diversity based on the corpus analysis proposed by Hoffman et al. (2013) in English is applicable to studies with Chinese. Thus we anticipate that the novel semantic variability measure based on the same method with some modifications should be able to be applied to studies in English. However, this would require further investigation.

In summary, the primary aim of this study was to investigate the effect of corpus-based ambiguity measures on Chinese character naming. We demonstrated convergent ambiguity effects based on using different approaches to address variation in meaning and contextual usage. Our measure SemVar can provide additional information about the substructure of the various contexts associated with a given word. Overall, these results provide evidence for the view that ambiguity of meaning is dependent on contextual variability.

## Electronic supplementary material

ESM 1 (XLSX 275 kb)

## Footnotes

1 We also tried a wider range of cluster solutions from 1 to 500, and the correlation between the two sets of scores was .998. The choice of the two ranges of cluster solutions was based on both the pilot observations and computational considerations. We could perhaps select to test a smaller range of contexts, but we would not be able to know the maximum in practice until we completed all of the analyses.

2 We used the promax rotation rather than other rotation methods (e.g., an orthogonal rotation) because the resulting loading data were understandable and easy to interpret. In addition, we made no assumption about whether the components should be orthogonal.
